# Recruiting Women to a Mobile Health Smoking Cessation Trial: Low- and No-Cost Strategies

**DOI:** 10.2196/resprot.7356

**Published:** 2017-11-03

**Authors:** Kristopher J Abbate, Melanie D Hingle, Julie Armin, Peter Giacobbi Jr, Judith S Gordon

**Affiliations:** ^1^ College of Medicine University of Arizona Tucson, AZ United States; ^2^ Department of Nutritional Sciences College of Agriculture & Life Sciences University of Arizona Tucson, AZ United States; ^3^ Department of Family and Community Medicine University of Arizona Tucson, AZ United States; ^4^ Department of Social and Behavioral Sciences West Virginia University Morgantown, WV United States; ^5^ College of Nursing University of Arizona Tucson, AZ United States

**Keywords:** smoking cessation, mobile applications, social media, women, mHealth

## Abstract

**Background:**

Successful recruitment of participants to mobile health (mHealth) studies presents unique challenges over in-person studies. It is important to identify recruitment strategies that maximize the limited recruitment resources available to researchers.

**Objective:**

The objective of this study was to describe a case study of a unique recruitment process used in a recent mHealth software app designed to increase smoking cessation among weight-concerned women smokers. The See Me Smoke-Free app was deployed to the Google Play Store (Alphabet, Inc., Google, LLC), where potential participants could download the app and enroll in the study. Users were invited in-app to participate in the study, with no in-person contact. The recruitment activities relied primarily on earned (free) and social media.

**Methods:**

To determine the relationship between recruitment activities and participant enrollment, the researchers explored trends in earned and social media activity in relation to app installations, examined social media messaging in relation to reach or impressions, and described app users’ self-reported referral source. The researchers collected and descriptively analyzed data regarding recruitment activities, social media audience, and app use during the 18-week recruitment period (March 30, 2015-July 31, 2015). Data were collected and aggregated from internal staff activity tracking documents and from Web-based data analytics software such as SumAll, Facebook Insights (Facebook, Inc.), and Google Analytics (Alphabet, Inc., Google, LLC).

**Results:**

Media coverage was documented across 75 publications and radio or television broadcasts, 35 of which were local, 39 national, and 1 international. The research team made 30 Facebook posts and 49 tweets, yielding 1821 reaches and 6336 impressions, respectively. From March 30, 2015 to July 31, 2015, 289 unique users downloaded the app, and 151 participants enrolled in the study.

**Conclusions:**

Research identifying effective online recruitment methods for mHealth studies remains minimal, and findings are inconsistent. We demonstrated how earned media can be leveraged to recruit women to an mHealth smoking cessation trial at low cost. Using earned media and leveraging social media allowed us to enroll 3 times the number of participants that we anticipated enrolling. The cost of earned media resides in the staff time required to manage it, particularly the regular interaction with social media. We recommend communication and cooperation with university public affairs and social media offices, as well as affiliate programs in journalism and communications, so that earned media can be used as a recruitment strategy for mHealth behavior change interventions. However, press releases are not always picked up by the media and should not be considered as a stand-alone method of recruitment. Careful consideration of an intervention’s broad appeal and how that translates into potential media interest is needed when including earned media as part of a comprehensive recruitment plan for mHealth research.

## Introduction

### Background

Since the introduction of the iPhone (Apple, Inc.) in 2007, integration of mobile and wireless technologies with everyday life has become ubiquitous, with broad reaching implications for health and health care. Tools and resources of varying quality for disease prevention and management previously limited by geography, cost, and time are now widely available and accessible via the Internet to the majority of individuals who seek these services. At the same time, an evidence base supporting the efficacy of mobile and wireless health behavior change interventions (mobile health, mHealth) has grown substantially as the field’s methodology and rigor continues to advance. However, foremost among remaining challenges is determining which mHealth intervention approaches work for specific conditions and outcomes. When, how, and for whom these approaches work best must also be elucidated. Critical to answering these questions is the ability of researchers to successfully recruit an adequate number of participants to mHealth trials.

There is a growing body of literature outlining best practices for participant recruitment [1,2], including use of social media. [3,4]. Nevertheless, such methods may not be entirely applicable to mHealth intervention studies. There are advantages and disadvantages when recruiting nationally for an mHealth study. The advantages include a much larger pool from which to draw, but the disadvantages include the potential for lack of contacts in the target community and no face-to-face interaction during which to build rapport and accountability. In addition, intervention studies may not be able to rely on services such as mTurk because of the inclusion or exclusion criteria, length of commitment, and other requirements for participating in an intervention, or depend on FindParticipants because of cost. Limited empirical data are available to inform approaches to recruiting participants to mHealth trials. Our own experience in conducting an mHealth trial highlighted several challenges in recruitment, including low return on investment of paid advertising [5]. Yet, recent polls show that large sectors of the population are actively using Web-based health resources [6] and wider demographics are seeking and connecting to social media communities, both of which hold promise for overcoming recruitment challenges in mHealth research [7].

Our systematic review of methods used to recruit participants to mHealth and Web-based interventions [8] identified several promising recruitment strategies specific to Web-based and mHealth studies, including search engine advertising, Web-based classifieds, paid advertising, and news stories (earned or free media) posted on social media. Eight out of 12 studies included in that review used some combination of social media, search term queries on multiple search engines, and health-focused websites to reach potential participants, whereas the remaining four studies used a combination of Web-based and traditional methods [8]. Each study targeted a specific population, and no two studies used identical recruitment methods. In addition, the recruitment periods and costs for each study varied widely. Therefore, we could not identify which strategy or combination of strategies were most effective or cost-effective [8]. Studies did not distinguish between participant yields from paid advertising versus other promotional efforts (ie, earned media). Given that researchers must decide how to invest limited recruitment resources, it is important to describe different methods of recruitment and their potential costs.

The authors conducted a study of the See Me Smoke-Free (SMSF) mHealth app to develop and evaluate the feasibility of the app designed to increase smoking cessation among women. SMSF specifically targeted women smokers because they face particular challenges when quitting [9]. Women gain an average of 9 pounds when quitting smoking [10-12], and weight gain is identified as a major reason for subsequent relapse [13]. In addition, women are disproportionately burdened by the health effects of smoking, with greater risks of cancer and coronary heart disease as compared with men [14,15].

The SMSF study was a within-subjects, pre- or posttest trial to develop and evaluate the feasibility of delivering a guided imagery intervention via a mobile phone mHealth app [16]. Study outcomes included feasibility of recruiting participants, retaining participants at the 30- and 90-day assessments, and adherence to the intervention [17]. We also explored the association between the use of the SMSF app and multiple health behaviors, including smoking cessation, healthy eating, increased physical activity, and improved body image [17]. The mHealth app was deployed to the Google Play Store. After download and completion of the user profile, users were invited to participate in a research study and consented within the app. Eligible participants were female, aged 18 years or older, spoke English, were US residents (to receive study incentives), smoked cigarettes in the past 30 days, and owned an Android (Alphabet, Inc., Google, LLC) phone (as the app was developed only for that platform). The intervention engaged participants in smoking cessation and behavior change using daily audio-guided mental imagery sessions initiated by the participant during scheduled sessions or when she felt an urge to smoke. The app included tracking and goal-setting features related to the number of tobacco-free days, servings of fruits and vegetables consumed, minutes spent engaged in physical activity, mood, and money saved by not smoking. SMSF also included links to additional information and resources, including a smoker’s quitline [9,16]. Participants were expected to use the app for most days during 1 month and to complete a survey at baseline, 30 days, and 90 days for which they were compensated a total of US $50. The University of Arizona’s institutional review board (IRB) deemed this research project to be exempt from oversight.

### Objectives

As part of this study, the research team developed a recruitment plan based on their literature review and previous experiences recruiting for electronic health or mHealth interventions. The plan included a stepwise approach to recruitment, starting with publicity through earned or social media and tobacco treatment contacts and then moving to paid advertising. First, the team partnered with the University of Arizona’s Office of Public Relations to issue a press release about the study. The resulting media exposure presented a unique opportunity to refocus our recruitment efforts to capitalize on this free media and leverage social media to recruit the target sample of 50 self-identified women smokers. Our sample size was determined to be sufficient to measure our feasibility goals (eg, achievement of our recruitment, enrollment, retention, and adherence), with a standard error of <7%, and to gather preliminary consumer satisfaction data [18]. In addition, with 50 participants and a significance level of .05, we had .80 power to detect change of *d*=0.66 (a medium effect size) in our cessation, diet, and physical activity outcomes. The purpose of this study was to describe this case study example, including our free media and social media recruitment efforts, and our results.

## Methods

### Overview

We present a case study describing the recruitment strategies used and results of these activities during our feasibility and acceptability study of the SMSF mHealth app. The feasibility study was conducted from January 2014 to December 2015. Recruitment of participants to the SMSF study occurred from March 30, 2015 to July 31, 2015, and analysis of the recruitment methods occurred from June 2016 to December 2016. Variables of interest included the number and timing of press releases and media mentions, Facebook reach, and Twitter (Twitter, Inc.) impressions. KA worked with JG to establish outcomes for the evaluation, which included descriptive analyses to explore trends in earned and social media activity in relation to app installations, examine social media messaging in relation to reach or impressions, and describe app users’ self-reported referral source. Data were collected from a variety of sources and assembled by KA to examine frequencies by date and content. The research assistant and KA tracked all study team posts to social media by date, frequency, content, and location. Press release distribution was tracked by date, media outlet, and content by the Office of Public Affairs (OPA). Media mention data by media outlet were sent from the OPA to the research assistant, who retrieved the coverage and entered the publication date, URL, content, and audience (local or national) into a spreadsheet. Twitter impressions were pulled from SumAll, a free software that tracks social media accounts, to examine frequencies and identify tweets yielding high impression data. Although the team managed a number of social media accounts, including Google+ and Pinterest, at the time, SumAll provided metrics for Facebook and Twitter only. KA pulled data regarding posts’ reach from Facebook Insights, examined frequencies, and identified specific posts with particularly high reach data. SMSF installation data were pulled from Google Analytics by date.

### Recruitment Activities and Timeline

During our previous mHealth study [5], we employed a recruitment strategy in which potential participants were invited to visit the research website to learn more about the study and if interested, enroll. Participants would then complete eligibility screening, informed consent, and the Web-based baseline survey. Once completed, they would receive an email with a link to download the app. This process was cumbersome and resulted in loss of participants before they downloaded the app. After several months, we changed our recruitment process so that the app was deployed to the App Store (the app was developed only for the iPhone operating system [iOS]) and recruited participants from within the app. This streamlined process resulted in a much higher conversion rate of potential participants to enrolled participants [5]. On the basis of this experience, we opted for the latter recruitment process in the SMSF study, and we deployed the SMSF app to the Google Play Store [19]. All recruitment activities occurred within the app after interested respondents downloaded it. After completing the SMSF profile, the app displayed a notification inviting the participant to enroll in the study (yes, no, and maybe). If the user selected “maybe,” she received a notification with more study description and another yes or no invitation. If the user answered “no,” she was returned to the SMSF home screen. If the user selected “yes,” she was taken to the in-app eligibility screener. If the user was not eligible, she was returned to the home screen. Eligible users began the in-app consent and baseline survey process. Additional details regarding enrollment can be found in the SMSF outcome paper [17].

Release of the app on the Google Play Store marked the beginning of the recruitment period (March 30, 2015). Learning from our previous experience with the high cost of Facebook and Google advertising for study recruitment [5], we had planned a multi-tiered recruitment strategy, including recruitment flyers in health care settings and cessation programs, soliciting volunteers on Craigslist, postings on social media sites, and paid Facebook advertising. As a first step, we worked with the University of Arizona Health Sciences’ Public Affairs Office to disseminate a press release about the study to the media and prominent members of the Arizona health care community. The purpose of the press release was to provide general information about the SMSF app and build credibility for the project that could be leveraged for other recruitment activities. The press release directed the reader to the Google Play Store or the project website which also linked directly to the Google Play Store (a copy of the press release is included in Multimedia Appendix 1). The first press release was sent to 191 local and Arizona media contacts (eg, *Arizona Daily Star* and *Cronkite News*). One week later, the app was released on the Play Store. Research team members also posted study information to 15 local digital media outlets with free classified listings.

The next phase of media releases described the SMSF app’s features, highlighted the app’s purpose, and focused on drawing women from throughout the United States—particularly from states with high smoking rates—to use the SMSF app. The second media release (April 28, 2015) targeted 17 publications that regularly feature articles on women’s health (eg, *Cosmopolitan* and *Harvard Women’s Health Watch*) and 66 outlets in US states with the highest smoking rates (eg, *The Columbus Dispatch* and *Lexington Herald Leader*). A study coinvestigator (PG) at West Virginia University (WVU) arranged for a third media release from his university’s Public Relations Office (May 12, 2015), which like the previous two project press releases described the SMSF app’s function and purpose but also highlighted his involvement as WVU faculty.

**Figure 1:**
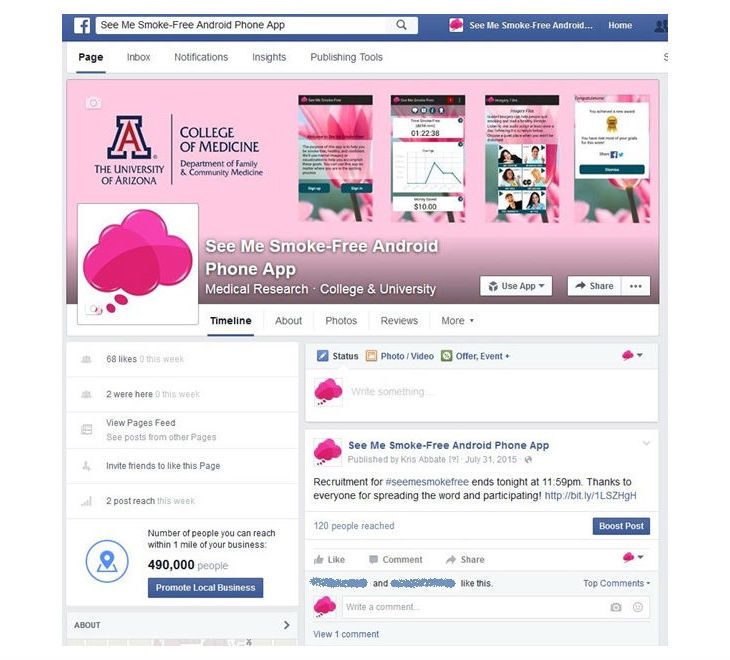
See Me Smoke-Free app Facebook profile page.

The research team disseminated earned media mentions (Web or printed references to SMSF not published by the study team) resulting from the press releases using the University of Arizona Facebook Family and Community Medicine (FCM) Tobacco Research Program and Twitter account, the SMSF Study Facebook page (Figure 1), and the study website connecting with broader social media campaigns on tobacco cessation whenever possible. The SMSF study team also identified and posted news articles and research studies about tobacco cessation on the study’s Facebook page to supplement social media outreach. For instance, the team posted a link to a study titled *Smoking and survival after breast cancer diagnosis in Japanese women: A prospective cohort study* to the SMSF Facebook page, accompanied by the caption, “More evidence that #smoking significantly increases #women’s risk of #breastcancer. (bitly link to article) #seemesmokefree helps women quit smoking, while also maintaining body weight and overall health (bitly link to SMSF website).” All of these posts leveraged media coverage of SMSF or related health issues by sharing the news in posts that directed the reader to the Google Play Store to learn more about and download the SMSF app. The vast majority of these efforts were performed by Mr Abbate, a medical student worker on the project, who spent on average 5 hours per week for 12 weeks on recruitment activities, including postings on social media. The total cost for his recruitment efforts was US $825 (60 hours @ US $13.75/hour [salary plus fringe]). During the remaining 4 weeks of recruitment, the research specialist performed these activities for a cost of approximately US $425 (20 hours @ US $21.25/hour [salary plus fringe]). Therefore, the total cost for these recruitment activities was US $1250 or US $8.28 per enrolled participant (N=151) and after accounting for participant attrition that occurred during the 3-month study period, US $17.12 per retained participant (N=73).

The use of hashtags with Facebook and Twitter postings ensured that these communications were included in search results of other postings focused on cessation or related topics. Posts that included more general hashtags (eg, #women) appeared to a large number of users searching social media for posts targeting women. Using a more health-specific hashtag with high relevance to women (eg, #breastcancer) was intended to further increase the likelihood that that SMSF posts appeared in the search results of users who were interested in health and inclined to participate in a research study. The research team also used hashtags associated with relevant and trending topics (eg, #NWHW indicated the established national campaign, National Women’s Health Week) that had the potential to be seen by large numbers of interested users. In this study, we used the hashtag #seemesmokefree to promote the study, which allowed researchers to easily follow SMSF-related postings and potential participants to follow the project and receive updates.

Finally, the research team also posted content to several private smoking cessation support groups on Facebook after receiving moderator approval. These groups had memberships ranging from 616 to 10,515 people (as of 28 July, 2015). All social media posts used IRB-approved language to describe the study and included a link to the SMSF study Web page.

### Data Capture and Analytics

Facebook reach (number of people whose Facebook feed contained SMSF content either in Facebook News Feed or in the right column ad area) and Twitter impressions (number of user feeds in which tweets and retweets appeared) data were used to document social media–related recruitment activity during recruitment. Google Analytics data were used to determine the number of daily page visits to the SMSF website and to document new app users—these data were exported and tabulated by date. Frequency and type (local, national, or international) of earned media coverage was monitored through personal contact with media outlets, especially Arizona State University’s Cronkite News Service and the Arizona Health Sciences’ Public Affairs Office, and automatically, using Google alerts. Finally, respondents who downloaded the app and opted to participate in the study (here defined as participants) answered several screening questions to determine their eligibility; among these questions was an optional multiple-choice item asking respondents to report how they learned about the SMSF study (ie, Facebook, Twitter, Pinterest, Google/+, Google Play Store, email, radio or television, newspaper, health care provider, friends or family, or other). Deidentified data were also collected on individuals who were confirmed app users but either did not qualify or opted out of the SMSF feasibility study (here defined as nonparticipants). All of these data were collected using the in-app questionnaire tool and tabulated.

## Results

### Media Mentions and Articles

**Figure 2:**
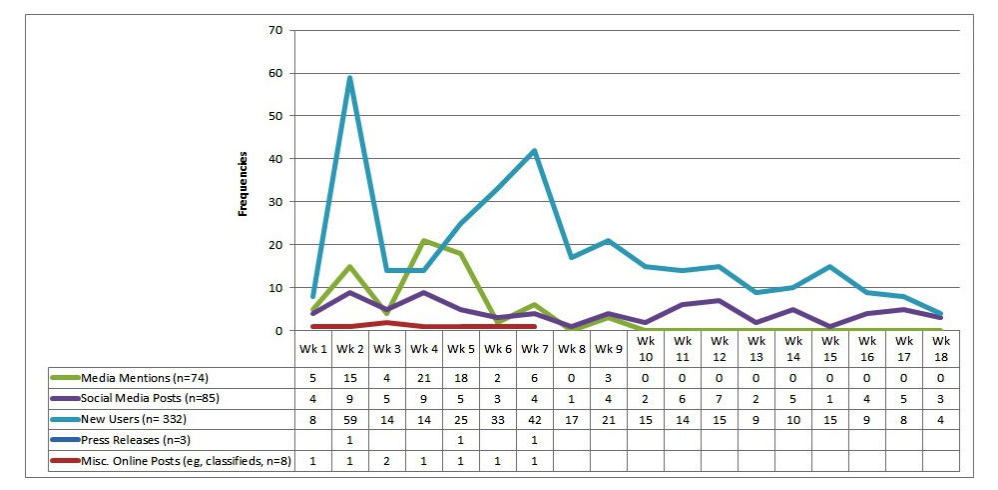
Recruitment activities and new users, March 30, 2015 to July 31, 2015.

Three press releases resulted in earned media mentions, including full-length printed or Web articles and broadcast pieces in 74 outlets: 34 local, 39 national, and 1 international. A summary of study recruitment activities relative to weekly recruitment outcomes is presented in Figure 2.

Media mentions included coverage by the Cronkite News - Phoenix Bureau, which distributed the story about SMFS nationwide through the Tribune News Service. This led to additional stories in the *Philadelphia Inquirer*, *Chicago Tribune*, *The Kansas City Star*, the *Idaho Statesman*, *The Modesto Bee*, and many others. After the initial press release, earned media mentions steadily accrued, beginning with a single mention on March 30 and concluding on May 27, with stories in two national publications. The most earned media mentions occurred on April 20 (10 local media outlets picked up one release) and April 28 (12 national outlets carried stories about the study). From April 1 to April 28 (weeks 1-5), 30 local media outlets and 15 national media outlets shared posts regarding the SMSF study.

**Figure 3:**
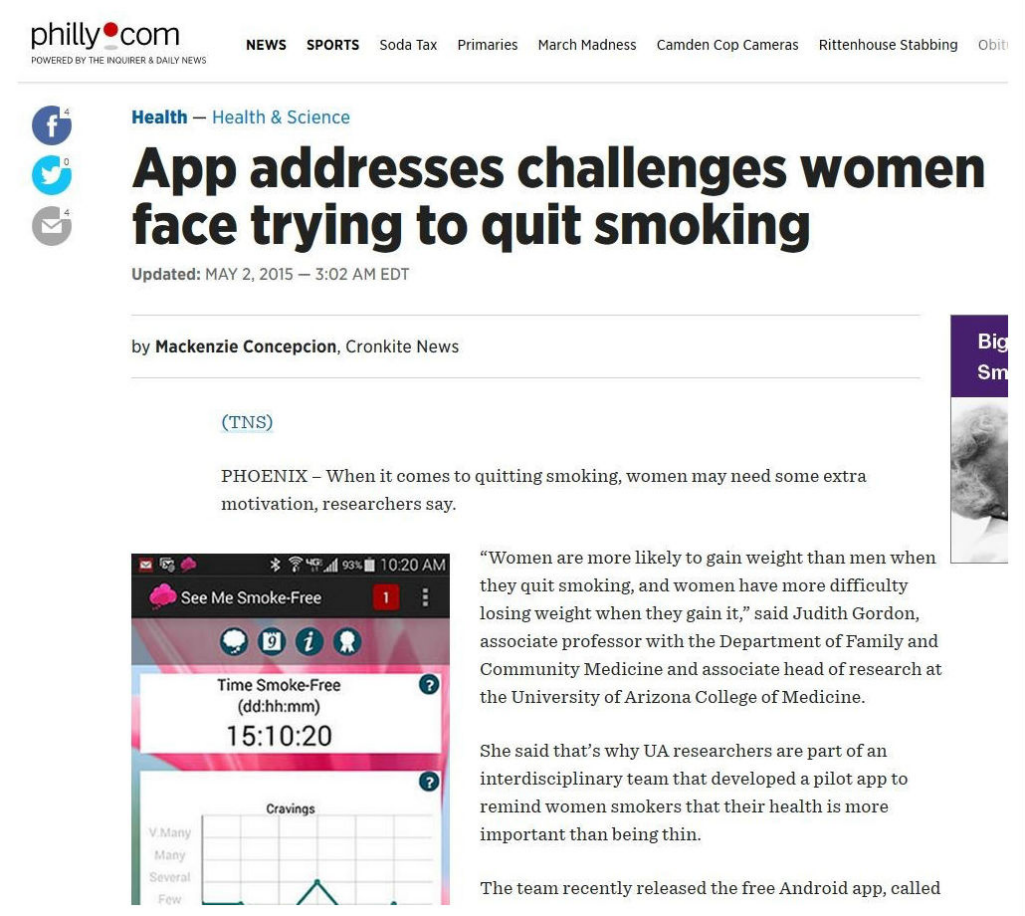
Article header screenshot.

Recruitment of participants occurred daily beginning on March 30 (week 1) until recruitment ended on July 31 (week 18). The most successful single days for recruitment occurred on April 9 (33 new users), May 11 (10 new users), and May 13 (12 new users).

As demonstrated in Figure 3, cross-platform sharing via email, Facebook, or Twitter expanded potential reach of Web-based coverage. The story, “App addresses challenges women face trying to quit smoking,” was shared 4 times each on Facebook and email. At the conclusion of recruitment, the SMSF Facebook page had 57 members, whereas the FCM Tobacco Twitter account had 200 followers. Because both accounts were public, posts could be viewed and accessed by users that were not direct followers, primarily through retweets, posts to third party groups, and social media platform search results.

### Social Media

During the recruitment period, the research team made 30 Facebook posts and 49 tweets, yielding 1821 reaches and 6336 impressions, respectively. This did not include posts made to three third-party private smoking cessation Facebook groups (one per group) whose respondent yields could not be documented. Four Facebook posts attained a reach of 100 or more each (see Table 1). All four posts were linked to earned media. Five Twitter posts resulted in at least 200 impressions each (as displayed in Table 2). Tweets using popular or woman-specific hashtags (eg, #NWHW, #women, and #healthyliving) and tagging popular accounts (eg, @Philadelphiagov and @cronkitenews) led to a greater number of impressions. Facebook posts were more detailed because they had no character limit, whereas tweets needed to be limited to 140 characters.

**Table 1:** Facebook post reach.

Description of Hook	Content	Reach
Link to a YouTube clip of a Cronkite News segment that included an interview with the study principal investigator	The interview link was accompanied by the following quote: “Among the challenges for women who want to stop smoking: they gain more weight on average when trying to quit. An app developed by University of Arizona researchers uses inspirational messages and other means to keep women committed to kicking the habit.”	April 22, 2015: 313 people reached
Link to a news segment that described the app on a local radio station (Mix 96.9, KFYI)	The segment was accompanied by the following caption: *“See Me Smoke Free* uses guided imagery, a technique that’s been used in sports and other disciplines for years. Motivational messages, a tracker of how long you’ve been smoke free, and other tools are available.”	May 4, 2015: 440 people reached
Link to an arkansasonline article	The article was posted with the following quote from the study principal investigator: “Most people smoke as a response to stress, and guided imagery may also help smokers quit by helping them relax. I’m not aware of any other app that’s used guided imagery to deal with the issue of stress related to smoking.”	November 5, 2015: 116 people reached
Link to a Maine News Online article	The article link was posted with a note that included an embedded link to the *See Me Smoke Free* Facebook page: “More coverage of the See Me Smoke-Free Android Phone App.”	May 27, 2015: 102 people reached

**Table 2:** Twitter post impressions.

Description of Hook	Content	Reach
Links to Cronkite News’ coverage of SMSF^a^ while tagging @cronkitenews	Arizona State University’s @cronkitenews covers See Me #smokefree app: *link*. #quitsmoking #healthyliving #news	April 21, 2015: 294 people reached
Links to SMSF study page in association with “NIH In Your State” campaign	NIH^b^-funded smoking cessation research in the @UofA Department of Family and Community Medicine *link* #NIHinYourState	May 6, 2015: 654 people reached
Links to the West Virginia University news article about SMSF collaboration with University of Arizona in association with National Women’s Health Week campaign	WVU’s Giacobbi collaborates with @UofA experts to launch “See Me Smoke-Free” app *link* #Android #NWHW #mHealth #smokefree	May 13, 2015: 225 people reached
Links to article about SMSF by local West Virginia TV affiliate WBOY	New #Android #App for #Women Aids #SmokeFree Quest	May 26, 2015, 205 people reached
Link to article about all time low smoking rates. Tags City of Philadelphia. Suggests mhealth, including SMSF may lower rates further	US cities such as @PhiladelphiaGov has all time low smoking rate. Can #mhealth #apps help this trend? *link* #seemesmokefree	June 29, 2015, 233 people reached

^a^SMSF: See Me Smoke-Free.

^b^NIH: National Institutes of Health.

### Referral Resources and Participant Demographics

**Figure 4:**
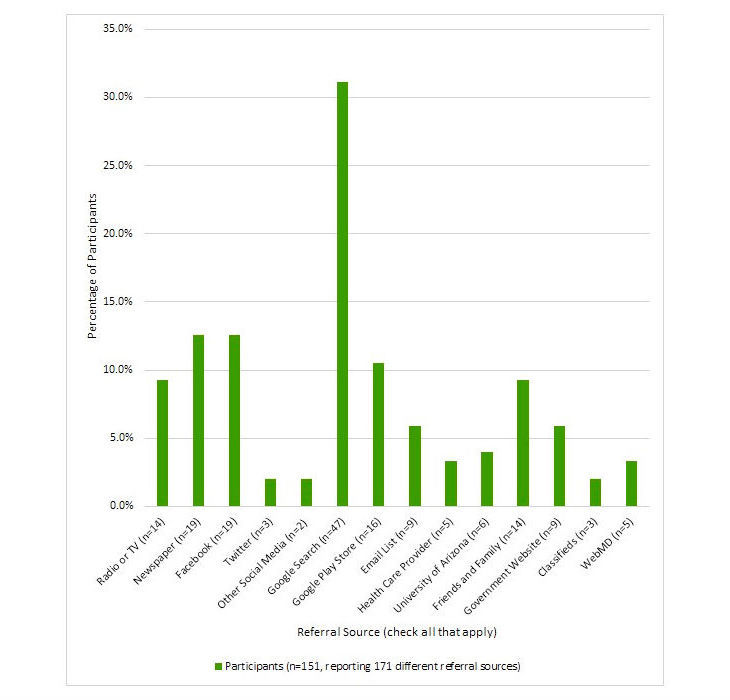
How participants became aware of the study.

SMSF app downloads yielded 289 users (defined as unique installations of the app) and 151 participants. Of the participants, 151 (100.0%) shared where they had heard about the SMSF through an in-app questionnaire. The participants reported a total of 171 referral resources across 14 categories (eg, radio or TV, newspaper, Facebook, and Twitter; Figure 4). Many participants reported learning about the study through multiple sources, with Google Search being the most endorsed referral source at 31.1% (47/151) followed by newspaper and Facebook at 12.6% (19/151) each.

As per the eligibility requirements, all participants were female. Participants were, on average, 39.1 (standard deviation 13.1) years of age, 72.5% (109/151) white, 65.7% (99/151) completed at least some college education, 57.5% (87/151) single, and 71.2% (108/151) “very dependent” on nicotine [17].

## Discussion

### Conclusions

No-cost recruitment efforts, via social media and in coordination with university public affairs offices that distributed press releases to media outlets, proved sufficient to secure adequate participant counts for the SMSF feasibility study. We were able to recruit 151 participants. We retained 73 participants throughout the study, and our baseline analyses indicated that there were no differences among those who remained in the study and those who dropped or withdrew. A more detailed discussion of retention can be found in the report of our feasibility outcomes [17]. Earned and social media approaches to recruitment have the potential to ensure socioeconomic, racial, and ethnic diversity in a study sample; however, certain populations may be less likely to use social media or to access Internet-based health news, although they are potential consumers of mHealth apps [20,21]. Our sample was representative of our target audience—US women smokers who use mHealth apps [22]—and was similar to other populations who use mHealth apps. Several studies have shown that mHealth users are predominantly white and have a relatively high level of education [23-26]. In addition, in the United States, mHealth users are primarily female [22-26]. Therefore, our sample appears to be representative of the population of women smokers who are most likely to use the SMSF program [6,26]. Future research is warranted to determine factors associated with lack of mHealth use and to elucidate strategies to attract more diverse populations to use mHealth apps.

Social media is increasingly a method by which to recruit research study participants, including studies focused on smoking cessation [27]. Unlike our study, which relied exclusively on earned media posts to social media, the majority of social media–based recruitment to date has relied upon targeted paid advertisements developed by the investigator and distributed by social networking companies through their marketing services [28,29].

The majority of our study participants reported learning of the SMSF app through a Google search, Facebook, or a newspaper. Nonparticipants indicated they learned of the SMSF app through TV, radio, or newspaper stories. Although we were not able to test for differences, these contrasting findings may suggest that our participants were actively seeking out methods for smoking cessation using Google and Facebook search engines, smoking-related stories or research, or joining a Facebook cessation group. It is also possible that Internet-savvy individuals were more likely to participate in a mobile app–based study. The fact that many participants reported hearing about the app through multiple sources not only indicates that information was disseminated through multiple channels but also that these channels are intertwined. For example, a hypothetical participant’s Google search may have yielded the SMSF Facebook page through which the participant accessed an earned media story.

The accumulation of app-related media mentions during the month of April did not result in a corresponding increase in users. This delay in app download and enrollment may have resulted from users feeling more comfortable joining a study when a specific media outlet or an accumulation of stories in multiple outlets contributed to credibility and legitimacy, or the fact that information about the app became widely available across multiple media outlets. This interpretation is supported by the number of users who reported hearing about the study from multiple platforms and media sources. The initial delay may also represent a period during which word-of-mouth sharing (from friend to friend, family member to family member, doctor to patient, etc) occurred. Although third-party sharing (eg, through an article, email, or social media sharing mechanisms) was beyond our ability to track, this may also explain steady accumulation of users even when new media mentions began to taper. Web publications and Web-based versions of radio and television broadcasts can be available indefinitely and accessed through a variety of methods (Internet search engine, direct referral, publication home page, etc). Social media posts advertising the end of recruitment may be responsible for a relative increase in new users during the second to last week of recruitment.

As recruitment progressed, we observed a decrease in the number of posts that received large numbers of reach and impressions on Facebook and Twitter. Facebook’s algorithms are designed to favor posts from major publishers while limiting meme-like posts, which would result in well-known content providers’ information to be displayed more frequently [29]. This suggests that content generated from mainstream media (ie, earned media stories), which is then shared via social media, has a greater chance of appearing in Facebook news feeds than organic posts by researchers to a study Facebook site. Additionally, the Facebook algorithms identify when fewer users engage with posts, and subsequent posts have less reach. Facebook encourages marketers to buy advertisements rather than relying on free posts [29]; indeed, Facebook posted a suggested advertisement layout to our study’s page moderator’s personal feed and recommended that the SMSF page would benefit from paid advertising. This also suggested that Facebook algorithms are not based on marketing intent (eg, recruitment to a research study vs advertisements for a commercial product or opportunity).

A frequent and unsubstantiated assertion in mHealth research is that online recruitment is more cost-effective than traditional approaches. However, as we experienced in our study, limited face-to-face interaction with respondents has the potential to slow or bias the recruitment process, and additional screening steps must be taken to ensure respondents are not bots or sham subjects whose study profiles were created solely for material gain. In a previous 3-month mobile app feasibility study that involved very little staff interaction with participants, we discovered sham subjects (eg, the same person signing up multiple times with different usernames and email addresses) when a research assistant attempted to reach participants by phone [5]. In the study described here, careful monitoring of participants’ study activities combined with phone and email communications with nonadherent participants enabled the research team to identify sham participants and drop them from the study. Building rapport and identifying and winnowing out sham subjects requires dedicated staff time and has implications for project budgets. Even when direct costs are not significant (eg, recruitment conducted using earned media), the distribution of earned media through websites, social media platforms, and email or texting necessitates staff time to manage inquiries and curate social media activity. At US $17.12 per participant (N=73), our cost was less than the US $43.29 per participant cost reported by Gilligan et al who used an earned and social media approach to recruit one-time survey completers [30]. Studies recruiting one-time survey completers via Facebook advertising have per-participant costs ranging from approximately US $4.00 [27,30] to US $25.00 [31,32], whereas research projects that require more participant time investment report higher Facebook advertising per-participant costs, up to approximately US $697 [33].

Although Facebook reach and Twitter impressions allow estimates of number of people who accessed pages or relevant posts, it was not possible to determine how many of those views represented our target demographic. With respect to Twitter and Facebook posts, it was also not possible to know whether posts were read, even when their presence was confirmed in potential subjects’ news feeds. Additionally, on at least one occasion, information was copied directly from an SMSF recruitment post and tweeted without attribution to the @FCMTobacco account, making it impossible to track how many Twitter users the information reached using Twitter’s analytics.

A large proportion of Web traffic involves the transfer of deidentified or encrypted data, which does not allow repeat impressions, reaches, and visits to be distinguished from new ones. Furthermore, self-report remains the only method of tracking certain referrals (eg, word-of-mouth and email and text message [short message service, SMS]–based referrals) and does not allow us to determine with any great degree of accuracy how respondents learned about the study. For example, respondents might have learned of the opportunity to participate through an earned media story on the radio but followed up with a Web search that brought them to the Google Play Store and allowed them to download the study app. It is possible that this respondent would self-report their recruitment as either “media story” or “Web search,” and both would be accurate. It is the nature of Web-based communication that information is disseminated and accessed in many different ways—ultimately all through a Web page or app—and that multiple strategies complement one another. In our study, earned media posted on social media were synergistic strategies that allowed us to earn credibility via a third party, which ultimately helped extend our reach in social media. Working with university public relations can further help position study-related stories and optimize the tone of the story for maximum appeal to a lay audience. However, we have attempted to use this strategy in other studies with less success. For example, a press release issued for an earlier study was not picked up by the media, and we had to rely on paid advertising for the majority of enrolled participants [5].

### Lessons Learned

Research regarding online recruitment methods for mHealth studies remains minimal, and findings are inconsistent. We demonstrated how earned media can be leveraged to recruit women to an mHealth smoking cessation trial at a low cost. The cost of earned media resides in the staff time required to manage it, particularly the regular interaction with social media. We recommend communication and cooperation with university public affairs and social media offices, as well as affiliate programs in journalism and communications, so that earned media can be used as a recruitment strategy for mHealth behavior change interventions. However, press releases are not always picked up by the media and should not be considered as a stand-alone method of recruitment. Careful consideration of an intervention’s broad appeal and how that translates into potential media interest is needed when including earned media as part of a comprehensive recruitment plan for mHealth research.

## Appendix

Multimedia Appendix 1 See Me Smoke-Free press release 4-21-2015.

## Abbreviations

FCM: Family and Community Medicine
IRB: institutional review board
mHealth: mobile health
NIH: National Institutes of Health
OPA: Office of Public Affairs
SMSF: See Me Smoke-Free
WVU: West Virginia University
