# Supplementary material for: Recruiting Women to a Mobile Health Smoking Cessation Trial: Low- and No-Cost Strategies
Source: JMIR Res Protoc. 2017 Nov 3;6(11):e219. doi: 10.2196/resprot.7356 (PMC5695655; doi:10.2196/resprot.7356)
Supplement: Multimedia Appendix 1 [file resprot_v6i11e219_app1.pdf]

THE UNIVERSITY OF ARIZONA
College of Medicine
Tucson

UA Researchers Seek Women Smokers to Test 'See Me Smoke-Free' Mobile App

National Cancer Institute-funded study to evaluate the first mobile app designed to help women quit smoking, eat well and get moving.

Contact: Jane Erikson, cell phone: 520-334-9464 April 28, 2015

MEDICAL WRITERS/ASSIGNMENT EDITORS NOTE: *Media are welcome to contact Dr. Judith Gordon directly at 520-626-6452 or judithg@email.arizona.edu*

TUCSON, Ariz. – Smoking results in more than 480,000 deaths each year, and smoking-related illness costs more than $300 billion in the United States, according to the U.S. Centers for Disease Control and Prevention.

Approximately 18 percent of U.S. adults are current smokers, and 11 states—Alaska, Arkansas, Kentucky, Louisiana, Mississippi, Missouri, Ohio, Oklahoma, South Carolina, Tennessee and West Virginia—have a smoking prevalence above the national average.

State	Smoking
**W. Virginia**	27.3%
**Kentucky**	26.5%
**Arkansas**	25.9%
**Mississippi**	24.8%
**Tennessee**	24.3%
**Oklahoma**	23.7%
**Louisiana**	23.5%
**Ohio**	23.4%
**Alaska**	22.6%
**Missouri**	22.1%
**S. Carolina**	22.0%

Prevalence of Smoking by State in 2013 – CDC

Of U.S. adults who smoke, the rate of smoking among women now is more than 15 percent, and women face specific challenges when quitting smoking.

To address those challenges, **See Me Smoke-Free**, the first multi-behavioral mobile health (mHealth) app designed to help women quit smoking, eat well and get moving, now is available for free on the Google Play Store. The app can be downloaded at https://play.google.com/store/apps/details?id=edu.arizona.guidedimagery

The See Me Smoke-Free research team is seeking participants from across the U.S. to test the app. Participants must be women smokers who use an Android smartphone. Additional information about the app and the research study is available at the website: www.seemesmokefree.org

Results of the study will be used to determine the feasibility of delivering a multi-behavioral intervention via a mobile app, assess users' satisfaction with the app, and estimate the effectiveness of the intervention in helping women to quit smoking. See Me Smoke-Free is funded by a two-year, $366,400 National Cancer Institute grant, 1R21CA174639.

The See Me Smoke-Free app is designed specifically for women, with input from women smokers, because studies have shown that women experience particular challenges when they quit smoking, like gaining weight, which may make quitting harder for them than for men, said **Judith S. Gordon, PhD**, who headed the multi-disciplinary research team that developed the app.

The goal of See Me Smoke-Free is to provide an overall sense of well-being and self-efficacy, said Dr. Gordon, associate professor and associate head for research with the **Department of Family and Community Medicine** at the **University of Arizona College of Medicine – Tucson**. "We want women to recognize that they are strong, they are beautiful, they are powerful and they're in control of their lives," she said. "And that they can use the app to engage in a healthier lifestyle."

"And that includes being smoke-free," she added.

The main component of the app is a guided imagery program, which consists of several audio files. Guided imagery is an enhanced visualization technique that encourages users to imagine themselves engaging in healthy behaviors, like being smoke-free, eating fresh fruits and vegetables, and being physically active. Guided imagery has long been used to improve athletic performance, and has been shown to help people quit smoking.

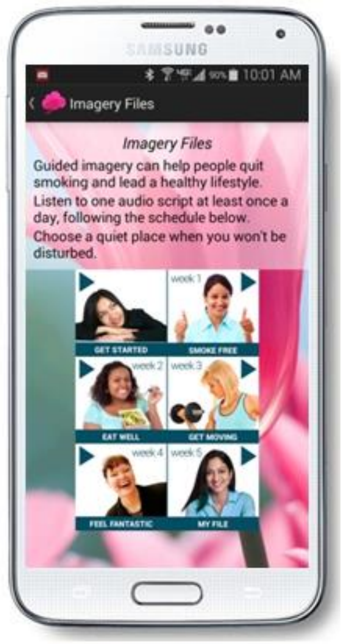

The See Me Smoke-Free app uses guided imagery to help women quit smoking, eat well and get moving.

In addition to sight imagery, the app prompts women to use all their senses for a fully immersive experience. For example, users are guided through a farmers' market, where they imagine seeing, smelling and tasting their favorite fruit or vegetable.

Users are prompted to use the guided imagery files daily. The app also allows users to access additional information and resources on quitting, eating well and being physically active; record achievement of their daily goals; and display how many days they have gone without smoking, the intensity of their cravings over time and how much money they have saved. Users will receive daily motivational messages and tips for living a healthy lifestyle, and will get virtual awards for meeting their goals and engaging with the app.

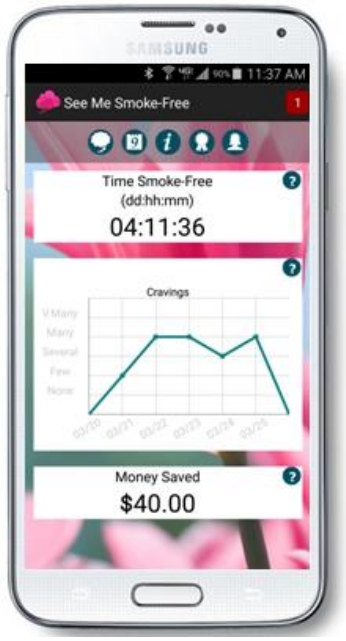

The See Me Smoke-Free app display includes how many days without smoking, cravings over time and money saved.

"A multi-behavioral intervention such as ours requires experts from a variety of fields," noted Dr. Gordon. The study team includes **Melanie Hingle, PhD, MPH, RD**, assistant professor with the Department of Nutritional Sciences, UA College of Agriculture and Life Sciences, and the Canyon Ranch Center for Prevention and Health Promotion at the UA Mel and Enid Zuckerman College of Public Health; **Thienne Johnson, PhD**, research associate with the Department of Electrical and Computer Engineering, UA College of Engineering, and the Department of Computer Science, UA College of Science; and **Peter Giacobbi, PhD**, associate professor with the College of Physical Activity and Sport Sciences and the School of Public Health at West Virginia University in Morgantown. **Jim Cunningham, PhD**, an epidemiologist with the UA Department of Family and Community Medicine, is the study's methodologist and statistician.

# # #
